# The Long-Term Public Health Impact of Social Distancing on Brain Health: Topical Review

**DOI:** 10.3390/ijerph18147307

**Published:** 2021-07-08

**Authors:** Anagha Kumar, Joel Salinas

**Affiliations:** 1 Harvard College, Harvard University, Cambridge, MA 02138, USA; anaghakumar@college.harvard.edu; 2 Center for Cognitive Neurology, Department of Neurology, New York University Grossman School of Medicine, New York University, New York, NY 10017, USA

**Keywords:** social isolation, cognitive health, brain health, loneliness, brain aging

## Abstract

Social distancing has been a critical public health measure for the COVID-19 pandemic, yet a long history of research strongly suggests that loneliness and social isolation play a major role in several cognitive health issues. What is the true severity and extent of risks involved and what are potential approaches to balance these competing risks? This review aimed to summarize the neurological context of social isolation and loneliness in population health and the long-term effects of social distancing as it relates to neurocognitive aging, health, and Alzheimer’s disease and related dementias. The full scope of the underlying causal mechanisms of social isolation and loneliness in humans remains unclear partly because its study is not amenable to randomized controlled trials; however, there are many detailed experimental and observational studies that may provide a hypothesis-generating theoretical framework to better understand the pathophysiology and underlying neurobiology. To address these challenges and inform future studies, we conducted a topical review of extant literature investigating associations of social isolation and loneliness with relevant biological, cognitive, and psychosocial outcomes, and provide recommendations on how to approach the need to fill key knowledge gaps in this important area of research.

## 1. Introduction

Social distancing has been a critical measure to address the COVID-19 pandemic, yet the long-term public health impact of prolonged social distancing is unclear. Although high levels of social isolation and loneliness have been associated with a high risk of morbidity and mortality Holt-Lunstad, Smith [1,2], a growing body of evidence strongly suggests that social isolation and loneliness—notable consequences of social distancing [3]—specifically play a major role in neurocognitive health [4,5,6,7]. Since the outbreak of COVID-19, research has further shown the increased dementia risk that may be due to stress, loneliness, and neuropsychiatric symptoms of prolonged physical distancing [8]. A key barrier to better understand the extent of potential long-term neurocognitive health impacts and to deploy effective strategies that balance competing risks requires clarifying underlying biological mechanisms. Investigating causal pathways that link social isolation and loneliness with neurocognitive aging and neuropathological changes is not readily amenable to be studied using randomized controlled trails. However, there are many detailed experimental models and observational studies that, when brought together, can provide a hypothesis-generating theoretical model for the complex neurobiology and pathophysiology underlying observed associations.

As indicated by the National Institute on Aging, there is a need for more research in the areas of social isolation and loneliness [9]. In response, this topical review was conducted to (1) highlight notable findings from previous studies related to potential biological mechanisms, (2) provide key recommendations to address critical knowledge gaps, and (3) inform future studies for addressing these factors in neurology and population health through targeted interventions. Although social isolation and loneliness may be manifestations of early neuropathological changes [10], the present review focused on summarizing the neurological context of social isolation and loneliness as proposed harmful psychosocial determinants of health and on describing the potential long-term risks of social distancing with respect to general neurocognitive health [11] and aging [12], inclusive of subsequent risk for developing Alzheimer’s disease and related disorders (ADRD) [13].

## 2. Definitions and Context

In the absence of well-established consensus definitions and research frameworks used across studies of social isolation and loneliness, we used generally accepted definitions for each to facilitate interpretation of extant literature. Social isolation was defined as the objective “lack of social contact or support” [14]. Separately, loneliness, which can arise from and compound social isolation, was defined as the subjective “feeling of being alone or isolated” [14] (i.e., a feeling that arises from a person perceiving less social support and connection than they desire or expect) [15]. We used these terms to explicitly indicate whether the noted outcomes were associated with loneliness, social isolation, or both.

Preceding the COVID-19 pandemic, loneliness and social isolation was rising in societies across the world; over one third of adults aged 45 and older are estimated to feel lonely, while about one fourth of adults aged 65 and older are likely to be socially isolated [16]. The COVID-19 pandemic’s required “social distancing” has had the unintended consequence of further increasing the prevalence of both social isolation and loneliness [17]. Due to the pandemic, individuals already at risk for loneliness (such as young adults, individuals with low household income, and adults living alone) have experienced higher levels of social isolation and loneliness during the COVID-19 pandemic [18]. Of note, social distancing may affect people differently depending on local, regional, and national conditions; thus, the interpretation and generalization of prior findings must be done cautiously and approaches to attenuate related risks must vary accordingly.

To summarize the potential causal neurobiological implications of social isolation and loneliness, this review focused on the suggested biological mechanisms between social isolation and neurological outcomes because causal relationships are best investigated through experimental and randomized controlled trials, which, in this case, are infeasible for human study. However, many animal studies have examined effects of social isolation, rather than loneliness, due to greater ease of controlling and measuring social interactions in animal models. Furthermore, since loneliness often emerges from social isolation [19], it is helpful to understand how—directly or indirectly—this exposure might alter brain biology, even without an associated measure of loneliness.

## 3. Methods

### 3.1. Literature Search

A literature search was performed in Embase, Cochrane Library, PubMed, MEDLINE, and ClinicalTrials.gov databases from their inception of each database (1966, 1946, 1974, 1966, and 2000 respectively) through March 2021. Search terms included: social isolation, loneliness, cognitive health, brain health, brain aging, cognitive aging, and dementia. Terms such as “Alzheimer’s disease” and “cognitive decline” were excluded because they fell under the hierarchy of the search terms used, which were the primary focus of the review. A filter was used to limit the search results to English language studies, and no filter was placed on publication dates. Studies were excluded if they did not relate social isolation or loneliness with measures that underly cognitive or brain health, cognitive or brain aging, or dementia due to any cause. The search terms were separately applied for each neurocognitive outcome as “loneliness and [outcome]” and “social isolation and [outcome]” in all databases. MEDLINE, ClinicaTrials.gov, and the Cochrane Library yielded no usable results, while PubMed and Embase delivered a total of 147 successful hits.

### 3.2. Eligibility Criteria

Studies were included if they reported examining the association of loneliness or social isolation with a neurocognitive outcome measure. Studies were excluded if they did not report a primary outcome of interest, did not evaluate loneliness or social isolation, or if it was a duplicate study. Intervention studies that were not designed to specifically decrease measures of loneliness or social isolation as their primary outcome, such as studies to improve psychosocial support for those with dementia, were also excluded. Non-English studies were excluded. We did not exclude studies based on patient demographics such as age, clinical status, or gender.

### 3.3. Data Extraction and Synthesis

The following data were extracted from the articles that met the inclusion criteria: study sample, control group type and format (for randomized controlled or experimental studies), outcome variables, and results for effects (see Supplementary Material). We also extracted any reported data on potential mechanisms of the results for effects, such as causal inference or mediation analyses. The results yielded studies that were either observational (cross-sectional, longitudinal) or experimental using animal models.

## 4. Discussion

**Figure 1:**
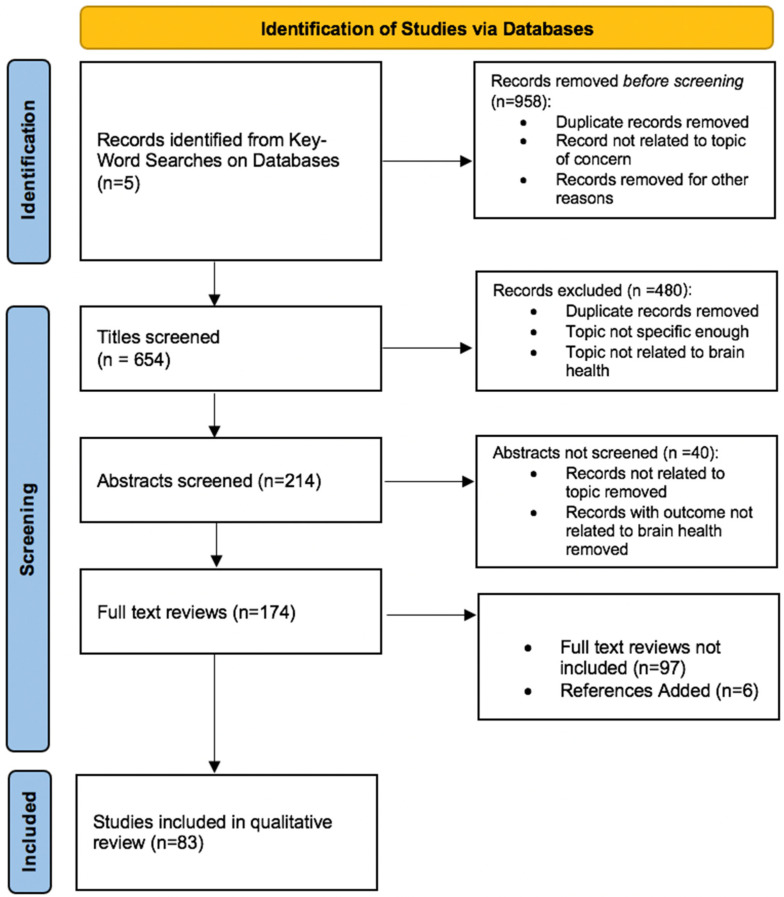
Derivation of literature reviewed: A literature search was conducted through key-word searches on databases that culminated in the inclusion of 83 studies in the qualitative review.

A total of 174 studies were reviewed. From a total of 1612 studies identified from databases, 1438 articles were excluded for reasons of not being related to the question of concern, being a duplicate, and concerning topics not related to brain health. After reviewing the full texts of 174 studies, 83 studies have been included in this qualitative review (Figure 1). Three key observations emerged from the review:

1. There are limited studies of biological mechanisms of social isolation and loneliness, but extant studies suggest that loneliness and social isolation are implicated in pathways that regulate experience-induced neuroplasticity as well as both systematic and brain-specific physiologic responses to stressors, such as inflammatory, neuroendocrine, or vascular dysfunction pathways; this is especially the case for environmental and psychosocial stressors that may be acute or chronic.
2. Among individuals who experience high levels of loneliness and social isolation, the combined effects of these pathways seem to be at least partly responsible for observed differences in vulnerability or resilience to cognitive decline due to aging or neuropathological changes.
3. Knowledge gaps to address, which emerged from the present review, are the need for additional studies that are designed to characterize promising stress and neuroplasticity-related pathways in humans, the need to identify how the influence of these pathways may vary by socioeconomic-based factors that differ for communities that have been historically discriminated against (e.g., access to resources and physical environments that promote positive social interactions and supportive social connections), and the need to explore practical intervention studies that target specific biological, cognitive, and psychosocial pathways in parallel.

**Figure 2:**
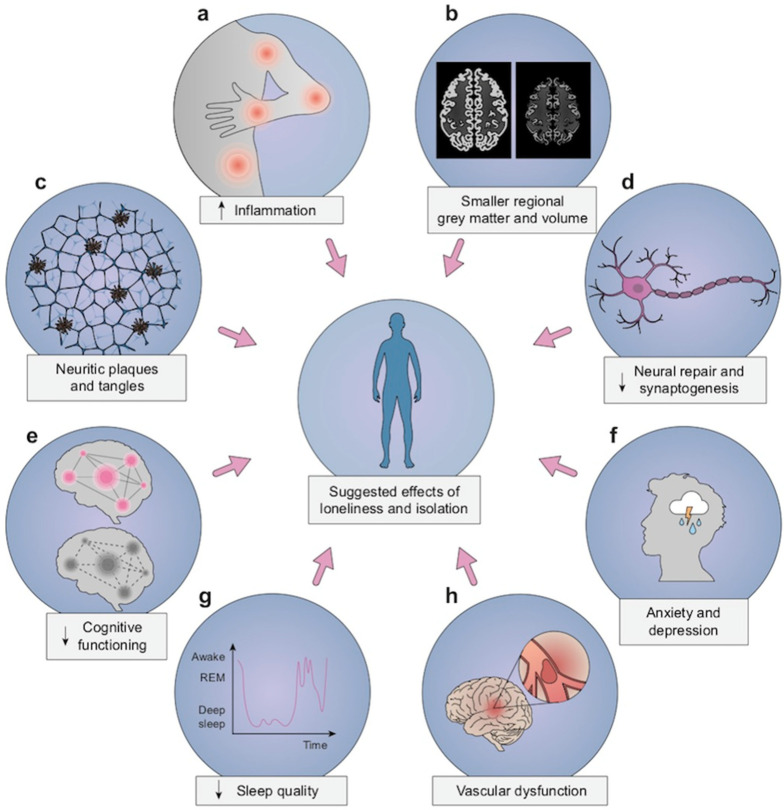
SIL has been related with (**a**) stress-dependent physiological disorders, chronic inflammation, and increased circulating markers for inflammation, (**b**) cortical atrophy and smaller regional brain volumes, including areas that support memory, (**c**) Alzheimer’s disease-related neuropathologic findings, such as amyloid plaques and neurofibrillary tangles, (**d**) decreased neurogenesis and lower levels of brain-derived neurotrophic factor, (**e**) increased risk of all-cause dementia and decreased levels of cognition function in executive function and memory domains, (**f**) poor mental health with increased symptoms of anxiety and depression, (**g**) worse sleep quality, especially sleep fragmentation, and (**h**) increased risk of cerebrovascular dysfunction and impaired post-stroke recovery.

Studies that investigated potential causal biological pathways were mostly derived from non-primate animal studies examining environmental conditions (i.e., enrichment versus isolation) due to the infeasibility of studying these causal models in humans. Almost all of the studies investigating associations with cognitive and psychosocial outcomes were human studies using measures of loneliness, social isolation, or both as predictor variables (Figure 2).

### 4.1. Biological Outcomes

#### 4.1.1. Associations with Inflammation

From the literature included in our qualitative review, social isolation and loneliness have been associated with serum markers of chronic inflammation, such as the neutrophil-to-lymphocyte ratio, the concentration of high-sensitivity C-reactive protein (CRP) [20], and circulating leukocytes [21]. Other circulating inflammatory markers, such as fibrinogen and interleukin-6 (IL-6), have been associated with increased levels of social isolation and loneliness as well [22,23]. In contrast, higher levels of social engagement and living with another individual were associated with lower levels of C-reactive protein and fibrinogen [24].

#### 4.1.2. Associations with Neuroimaging Measures

Of the studies reviewed, most identified that loneliness or social isolation related with brain MRI measures of brain aging and neuropathology. For example, those who identify with feeling lonely were shown to have smaller hippocampal volumes and a larger volume of cerebral white matter implicated in social cognitive processing and emotional regulation [25]. Regions such as the left posterior superior temporal sulcus, the middle temporal gyrus, and the entorhinal cortex (involved in social perception and associative memory) have also been found to be smaller among participants who had smaller online social networks [26,27]. Furthermore, based on tau PET imaging, it has been found that higher tau pathology in the right entorhinal cortex and clusters in the right fusiform gyrus are associated with greater loneliness [28]. Higher cortical amyloid burden on PET scans has also been shown to be significantly associated with greater loneliness [10]. In addition, lonely individuals have shown stronger functional communication in the default network and higher microstructural integrity in the fornix pathway of the default network in a functional connectivity MRI [29]. Overall, the causal directionality and etiology of these neuroanatomical differences remains unclear.

#### 4.1.3. Associations with Neuropathology

Of the studies included in this narrative review, Alzheimer’s disease pathology, including greater burden of amyloid plaques and neurofibrillary tau tangles, have been associated with greater loneliness even after controlling for other markers such as age, sex, and apolipoprotein E ε4, the genetic risk marker of Alzheimer’s disease [10,28,30]. Moreover, the association of high amyloid burden and loneliness has been shown to be stronger in APOE ε4 carriers than in noncarriers, indicating that individuals with genetic risk for Alzheimer’s disease may be at greater risk of loneliness and social isolation [10]. Social network size has also been proven to act as a modifier of association between pathology and cognitive function [31]. For dementia-related neuropathology in the form of greater cerebrovascular disease burden, participants within a smaller social network (and thus greater social isolation) have been shown to be at higher risk of ischemic stroke [32,33].

#### 4.1.4. Associations with Neuroplasticity

Social isolation and loneliness have been implicated in neuroplasticity related to post-stroke recovery and vascular health in the literature reviewed. In two mouse models of ischemic stroke, social isolation immediately following cerebral ischemia (for 15 days and 8 and 90 days respectively) was related with greater brain volume loss, higher mortality, delayed motor and sensory recovery, and worsened cognitive function [34,35]. Mice isolated immediately after stroke have shown brain tissue with decreased levels of brain-derived neurotrophic factor (BDNF), a molecule that aids in synaptogenesis and the growth, repair, and maturation of neuronal cells [34,35]. Similarly, group-living animals when socially isolated have been shown to have a decrease in cell proliferation, specifically in the dentate gyrus [36,37]; enriched social environments have been shown to increase cell proliferation and neurogenesis, especially in regions implicated in social interaction, memory, and communication [38,39,40,41,42,43]. A large literature of social animals randomly assigned to normal social living conditions or socially isolated conditions have also indicated the correlation of social isolation to low neurogenesis, BDNF, nerve growth factor (involved in growth and maturation of neurons as well), and low cell proliferation in the amygdala [40,41,44]. Early instances of social isolation in rats have been shown to affect the development of cognitive abilities and the nervous system through the mediation of producing BDNF protein [45]. Adolescent social isolation has correlated with epigenetic modifications, in the form of acetylation, that affect the expression of the BDNF in which isolation-reared rats have shown decreased hippocampal BDNF mRNA levels and protein expression. Thus, social isolation may play a causal role in the production of BDNF through gene regulation pathways, such as epigenetic modifications [45]. Social interaction has also been shown to rescue memory impairment in an Alzheimer’s disease mouse model through a hippocampal BDNF-mediated pathway [38]. Even though BDNF levels measured from neural tissue is a more reliable indicator of this brain-enriched pathway, serum BDNF levels in humans have also been shown to partly mediate the association between levels of social support and dementia risk [46].

#### 4.1.5. Associations with Sleep

From the literature reviewed, in both older adults and adolescents, loneliness and social isolation have been associated with worse sleep quality, typically in the form of sleep fragmentation [47,48,49,50,51,52]. Poor sleep quality has also been linked with a higher risk of cognitive decline and poorer neurocognitive health [53,54]. Although the directionality of these relationships is unclear, one study of older adults suggested that loneliness mediated the association between sleep and psychosocial health [55,56].

### 4.2. Cognitive Outcomes

#### Associations with Cognitive Function

Of the studies reviewed, low levels of social isolation (either from high social activity or large social networks) have been associated with better late-life cognitive functioning [4]. Inversely, social isolation has been associated with worse executive functioning [57,58] and memory loss [59,60]. In rodent models, social isolation decreased the activity of cAMP response element-binding protein (CREB), a transcription factor involved in long-term memory formation [61]. Furthermore, feeling lonely has been associated with an increased risk of all-cause mild cognitive impairment [10,62,63]; social isolation has been associated with global cognitive decline in older adults [64]. Loneliness has also correlated with poorer healthcare and financial decision making in older adults, further implicating executive functioning as a cognitive domain particularly vulnerable to impairment in the setting of loneliness [65]. Although longitudinal studies cannot establish causal directionality, these studies consistently demonstrate an association between loneliness and subsequent cognitive decline [63]. Loneliness has reliably been associated with increased risk of Alzheimer’s disease and related dementias [66,67,68,69], in addition to worse behavioral and psychological symptoms of dementia [70]. Moreover, this increase in dementia risk seems to be most relevant for individuals with persistent loneliness compared to individuals with transient loneliness in whom dementia risk remains unchanged [67].

### 4.3. Psychosocial Outcomes

#### 4.3.1. Associations with Depression, Anxiety, and Stress

During the COVID-19 pandemic, isolated older adults reported higher levels of depressive symptoms, specifically worse social well-being and greater loneliness [71]. In studies reviewed, higher levels of loneliness have historically been associated with increased levels of depression and anxiety [72,73]. Though, in addition to psychiatric symptoms, social isolation and loneliness have been linked with related stress-dependent physiological changes [74]. Individuals who tend to be more lonely, including adolescents, are likely to also experience higher levels of social stress [75]. Furthermore, loneliness has been associated with higher levels of stress hormones that are typically elevated in response to psychosocial stressors [76]. In animal studies, chronic stress from social isolation has been shown to affect group-living animals by triggering anxiety-like behavior. At the molecular level, oxytocin expression and oxytocin-Ca^2+^ signaling proteins, which are important for socioemotional and executive functioning, were permanently decreased in the hypothalamus, hippocampus, and prefrontal cortex [74]. Loneliness has been tied to increased hypothalamic pituitary adrenocortical (HPA) activity, mediating corticosteroid production [57,77,78,79], which may also increase risk of depression, anxiety, neurodegeneration, and immune and metabolic disorders [80,81,82]. Similarly, evidence suggests chronic loneliness associates with decreased variance in cortisol levels across the waking day as a biological measure of chronic stress [83].

#### 4.3.2. Mediating and Modifying Factors

Eleven studies investigated sociodemographic factors as potential mediators in the association between loneliness and neurocognitive health. These factors include age, gender, socioeconomic status, and race/ethnicity. Loneliness has closely related with the mental health of both adolescents [84] and older adults [85], though older adults are uniquely susceptible to observed relationships between loneliness and increased risks of physical inactivity, cognitive impairment, fatigue, and impaired activities of daily living [86].

Because loneliness is more prevalent among women [87], gender has been a proposed modifier of the relationship between loneliness and neurocognitive health. Older women are also more likely to be socially isolated based on census data demonstrating that, compared to men, women live alone more frequently [34]. In addition to higher risk for exposure to social isolation and loneliness in women, animal models suggest long-standing sex-dependent effects on abnormal gene expression in the brain related to neurological dysfunction [74,88,89].

While gender and age likely play a modifying role in health and disease mechanisms that relate with loneliness and social isolation, to the best of our knowledge fewer studies (1 of 174 studies reviewed) have been published that were specifically designed from inception to analyze how race/ethnicity play a role in the complex associations of loneliness and social isolation with neurocognitive health. After adjusting for differences in income, employment status, depressive symptoms, and social network size, compared to White older adults, lonely Black older adults had higher risk of dementia and cognitive impairment [90].

### 4.4. Future Directions

#### 4.4.1. Intervention Studies

More intervention studies are needed to identify pragmatic, cost-effective strategies that decrease or prevent social isolation and loneliness and can attenuate their associated risks. For the present review, two intervention studies were identified. Examples of existing evidence-based interventions include community initiatives and social prescribing, or establishing a link between health services and social interventions to improve well-being [91]. Other interventions identified in a metanalysis targeted the improvement of social skills, enhancing social support, increasing occasions for social contact, and addressing maladaptive social cognition [91,92]. Successful interventions that reduce levels of loneliness are centered around promoting a sense of belonging and social connectedness [91], which tend to use methods tailored to address the precise barriers that underly an individual’s or a group’s social isolation or loneliness. For instance, some individuals lack social connectedness due to government orders to socially distance and stay home, and other individuals experience loneliness or social isolation due to a lack of mobility, untreated mood disorder that limits frequency and quality of social interactions, or a lack of skills in interpersonal effectiveness to meet their desire for greater social contact and support. Similarly, some older adults may lack the access or knowledge to use technologies that facilitate virtual or in-person opportunities for interaction. For older adults, interventions have revolved around physical exercise [93], social activities [94], or a mix of these [95]. Importantly, despite examples of significant findings with notable magnitude of effects, there are a limited number of community-based intervention studies compared to the more common primary care and home-based interventions [92]. New research may benefit from recruiting and training members of the community to create and perpetuate intergenerational companionships aimed at reducing the burden of loneliness and social isolation in older adults. Other possible interventions may explore brief yet effective pragmatic strategies to promote social support or rapid screening methods to assess loneliness and social isolation.

#### 4.4.2. Recommendations to Address Knowledge Gaps

First, given existing gaps in understanding how social isolation and loneliness both modify and mediate pathways of experience-induced neuroplasticity and physiological responses to environmental and psychical stressors, studies to clarify specific mechanisms and pathways concerning inflammatory, neuroendocrine, and vascular dysfunction pathways can help to remediate the implications of broken or small social networks. Characterizing the intricacies of the effects of social isolation and loneliness on these biological pathways—through experimental and observational studies—will inform strategies that are better targeted to reverse such effects through medical or psychosocial interventions, or both.

Second, additional studies are needed that not only characterize the pathways of impact in the human brain, but also identify how these socioeconomic-based factors may compound these pathways. Future longitudinal studies designed to clarify the role that race and ethnicity plays in the effect of social isolation and loneliness on brain health are required to develop successful public health interventions.

Third, policy initiatives to support this research and that promote and enhance supportive social connections should be prioritized. This may include supporting and further highlighting the need for psychosocial intervention studies or efforts to increase technology and internet access for older adults. After the pandemic subsides, the long-term impact of increased levels of loneliness and social isolation will remain to be seen, but building on prior work to address the important knowledge gaps recommended above creates a valuable opportunity to radically improve public health and well-being in the decades to come. As a result, it is critical that all members of the community, from healthcare workers to politicians to scientists, come together to address the potential consequences of social isolation and loneliness.

## 5. Conclusions

Prioritizing studies that identify and characterize the most critical pathways that underlie the associations of social isolation and loneliness with neurocognitive health and disease states is of high importance, because insights from this research will inform better intervention trials. This includes adequately accounting for relevant factors that may both alter risk and advance health equity (such as age, gender, race/ethnicity, and socioeconomic status). Knowledge gaps in this area of research must be addressed to better understand the long-terms risks and benefits of public health interventions that are likely to influence levels of social isolation and loneliness within our communities.

## Supplementary Materials

The table of articles included in the review are available online at https://www.mdpi.com/article/10.3390/ijerph18147307/s1. Table S1: Summary of Literature Review.

## References

[1] Holt-Lunstad J., Smith T.B., Layton J.B. (2010). Social Relationships and Mortality Risk: A Meta-analytic Review. PLoS Med..

[2] Luo Y., Hawkley L.C., Waite L.J., Cacioppo J.T. (2012). Loneliness, health, and mortality in old age: A national longitudinal study. Soc. Sci. Med..

[3] McGinty E.E., Presskreischer R., Han H., Barry C.L. (2020). Psychological Distress and Loneliness Reported by US Adults in 2018 and April 2020. JAMA.

[4] Evans I.E., Martyr A., Collins R., Brayne C., Clare L. (2019). Social Isolation and Cognitive Function in Later Life: A Systematic Review and Meta-Analysis. J. Alzheimer Dis..

[5] Okamoto S., Kobayashi E. (2020). Social Isolation and Cognitive Functioning: A Quasi-Experimental Approach. J. Gerontol. Ser. B.

[6] Cacioppo J.T., Cacioppo S. (2014). Social Relationships and Health: The Toxic Effects of Perceived Social Isolation. Soc. Personal. Psychol. Compass.

[7] Clair R., Gordon M., Kroon M., Reilly C. (2021). The effects of social isolation on well-being and life satisfaction during pandemic. Humanit. Soc. Sci. Commun..

[8] Livingston G., Huntley J., Sommerland A., Ames D., Ballard C., Banerjee S., Brayne C., Burns A., Cohen-Mansfield J., Cooper C. (2020). Dementia prevention, intervention, and care: 2020 report of the Lancet Commission. Lancet.

[9] Necka E. (2021). After COVID, Research on Social Isolation and Loneliness Is Needed More than Ever.

[10] Donovan N.J., Okereke O.I., Vannini P., Amariglio R.E., Rentz D.M., Marshall G.A., Johnson K.A., Sperling R.A. (2016). Association of Higher Cortical Amyloid Burden With Loneliness in Cognitively Normal Older Adults. JAMA Psychiatry.

[11] Poey J.L., Burr J.A., Roberts J.S. (2017). Social Connectedness, Perceived Isolation, and Dementia: Does the Social Environment Moderate the Relationship Between Genetic Risk and Cognitive Well-Being?. Gerontologist.

[12] Cudjoe T.K.M., Roth D.L., Szanton S.L., Wolff J.L., Boyd C.M., Thorpe R.J. (2020). The Epidemiology of Social Isolation: National Health and Aging Trends Study. J. Gerontol. Ser. B.

[13] Holwerda T.J., Deeg D.J.H., Beekman A.T.F., van Tilburg T., Stek M.L., Jonker C., Schoevers R.A. (2014). Feelings of loneliness, but not social isolation, predict dementia onset: Results from the Amsterdam Study of the Elderly (AMSTEL). J. Neurol. Neurosurg. Psychiatry.

[14] Veazie S., Gilbert J., Winchell K., Paynter R., Guise J.-M. (2019). Addessing Social Isolation to Improve the Health of Older Adults: A Rapid Review.

[15] Wang J., Mann F., Lloyd-Evans B., Ma R., Johnson S. (2018). Associations between loneliness and perceived social support and outcomes of mental health problems: A systematic review. BMC Psychiatry.

[16] National Academies of Sciences, Engineering, and Medicine (2020). Social Isolation and Loneliness in Older Adults: Opportunities for the Health Care System.

[17] Rozenkrantz L., Bernstein M.H., Hemond C.C. (2020). A paradox of social distancing for SARS-CoV-2: Loneliness and heightened immunological risk. Mol. Psychiatry.

[18] Bu F., Steptoe A., Fancourt D. (2020). Loneliness during a strict lockdown: Trajectories and predictors during the COVID-19 pandemic in 38,217 United Kingdom adults. Soc. Sci. Med..

[19] Rook K.S. (1984). Research on Social Support, Loneliness, and Social Isolation: Toward an Integration. Rev. Personal. Soc. Psychol..

[20] Koyama Y., Nawa N., Yamaoka Y., Nishimura H., Sonoda S., Kuramochi J., Miyazaki Y., Fujiwara T. (2021). Interplay between social isolation and loneliness and chronic systemic inflammation during the COVID-19 pandemic in Japan: Results from U-CORONA study. Brain Behav. Immun..

[21] Cole S.W., Hawkley L.C., Arevalo J.M., Sung C.Y., Rose R.M., Cacioppo J.T. (2007). Social regulation of gene expression in human leukocytes. Genome Biol..

[22] Smith K.J., Gavey S., Riddell N.E., Kontari P., Victor C. (2020). The association between loneliness, social isolation and inflammation: A systematic review and meta-analysis. Neurosci. Biobehav. Rev..

[23] Vingeliene S., Hiyoshi A., Lentjes M., Fall K., Montgomery S. (2019). Longitudinal analysis of loneliness and inflammation at older ages: English longitudinal study of ageing. Psychoneuroendocrinology.

[24] Walker E., Ploubidis G., Fancourt D. (2019). Social engagement and loneliness are differentially associated with neuro-immune markers in older age: Time-varying associations from the English Longitudinal Study of Ageing. Brain Behav. Immun..

[25] Lind A., Salomäki S., Parkkola R., Haataja L., Rautava P., Junttila A., Koikkalainen J., Lötjönen J., Saunavaara V., Korja R. (2020). Brain volumes in relation to loneliness and social competence in preadolescents born very preterm. Brain Behav..

[26] Kanai R., Bahrami B., Duchaine B., Janik A., Banissy M.J., Rees G. (2012). Brain Structure Links Loneliness to Social Perception. Curr. Biol..

[27] Kanai R., Bahrami B., Roylance R., Rees G. (2012). Online social network size is reflected in human brain structure. Proc. R. Soc. B Biol. Sci..

[28] Uquillas F.D., Jacobs H.I.L., Biddle K.D., Properzi M., Hanseeuw B., Schultz A.P., Rentz D.M., Johnson K.A., Sperling R.A., Donovan N.J. (2018). Regional tau pathology and loneliness in cognitively normal older adults. Transl. Psychiatry.

[29] Spreng R.N., Dimas E., Mwilambwe-Tshilobo L., Dagher A., Koellinger P., Nave G., Ong A., Kernbach J.M., Wiecki T.V., Ge T. (2020). The default network of the human brain is associated with perceived social isolation. Nat. Commun..

[30] Dong H., Goico B., Martin M., Csernansky C., Bertchume A., Csernansky J., Dong H., Goico B., Martin M., Csernansky C. (2004). Modulation of hippocampal cell proliferation, memory, and amyloid plaque deposition in APPsw (Tg2576) mutant mice by isolation stress. Neuroscience.

[31] Bennett D.A., Schneider J.A., Tang Y., Arnold S.E., Wilson R.S. (2006). The effect of social networks on the relation between Alzheimer’s disease pathology and level of cognitive function in old people: A longitudinal cohort study. Lancet Neurol..

[32] Hankey G.J. (2014). Social Network and Stroke Risk. Stroke.

[33] Nagayoshi M., Everson-Rose S., Iso H., Mosley T.H., Rose K.M., Lutsey P.L. (2014). Social Network, Social Support, and Risk of Incident Stroke. Stroke.

[34] Holmes A., Xu Y., Lee J., Maniskas M.E., Zhu L., McCullough L.D., Venna V.R. (2020). Post-Stroke Social Isolation Reduces Cell Proliferation in the Dentate Gyrus and Alters miRNA Profiles in the Aged Female Mice Brain. Int. J. Mol. Sci..

[35] Venna V.R., Xu Y., Doran S., Patrizz A., McCullough L.D. (2014). Social interaction plays a critical role in neurogenesis and recovery after stroke. Transl. Psychiatry.

[36] Van Praag H., Kempermann G., Gage F.H. (2000). Neural consequences of enviromental enrichment. Nat. Rev. Neurosci..

[37] Gheusi G., Ortega-Perez I., Murray K., Lledo P.-M. (2009). A niche for adult neurogenesis in social behavior. Behav. Brain Res..

[38] Hsiao Y.-H., Hung H.-C., Chen S.-H., Gean P.-W. (2014). Social Interaction Rescues Memory Deficit in an Animal Model of Alzheimer’s Disease by Increasing BDNF-Dependent Hippocampal Neurogenesis. J. Neurosci..

[39] Dunlap K.D., Chung M. (2013). Social novelty enhances brain cell proliferation, cell survival, and chirp production in an electric fish, Apteronotus leptorhynchus. Dev. Neurobiol..

[40] Dunlap K.D., Chung M., Castellano J.F. (2013). Influence of long-term social interaction on chirping behavior, steroid levels and neurogenesis in weakly electric fish. J. Exp. Biol..

[41] Dunlap K.D., Silva A.C., Chung M. (2011). Environmental complexity, seasonality and brain cell proliferation in a weakly electric fish, Brachyhypopomus gauderio. J. Exp. Biol..

[42] Lieberwirth C., Wang Z. (2012). The Social Environment and Neurogenesis in the Adult Mammalian Brain. Front. Hum. Neurosci..

[43] Zupanc G.K.H., Sîrbulescu R.F. (2011). Adult neurogenesis and neuronal regeneration in the central nervous system of teleost fish. Eur. J. Neurosci..

[44] Westenbroek C., Boer J.A.D., Veenhuis M., Ter Horst G.J. (2004). Chronic stress and social housing differentially affect neurogenesis in male and female rats. Brain Res. Bull..

[45] Li M., Du W., Shao F., Wang W. (2016). Cognitive dysfunction and epigenetic alterations of the BDNF gene are induced by social isolation during early adolescence. Behav. Brain Res..

[46] Salinas J., Beiser A., Himali J.J., Satizabal C.L., Aparicio H., Weinstein G., Mateen F.J., Berkman L.F., Rosand J., Seshadri S. (2017). Associations between social relationship measures, serum brain-derived neurotrophic factor, and risk of stroke and dementia. Alzheimer Dement. Transl. Res. Clin. Interv..

[47] Benson J.A., McSorley V.E., Hawkley L.C., Lauderdale D.S. (2021). Associations of loneliness and social isolation with actigraph and self-reported sleep quality in a national sample of older adults. Sleep.

[48] Eccles A.M., Qualter P., Madsen K.R., Holstein B. (2020). Loneliness in the lives of Danish adolescents: Associations with health and sleep. Scand. J. Public Health.

[49] Cacioppo J.T., Hawkley L.C., Berntson G.G., Ernst J.M., Gibbs A.C., Stickgold R., Hobson J.A. (2002). Do Lonely Days Invade the Nights? Potential Social Modulation of Sleep Efficiency. Psychol. Sci..

[50] Hawkley L.C., Preacher K.J., Cacioppo J.T. (2010). Loneliness impairs daytime functioning but not sleep duration. Health Psychol..

[51] Jacobs J.M., Cohen A., Hammerman-Rozenberg R., Stessman J. (2006). Global Sleep Satisfaction of Older People: The Jerusalem Cohort Study. J. Am. Geriatr. Soc..

[52] Kurina L.M., Knutson K., Hawkley L.C., Cacioppo J.T., Lauderdale D.S., Ober C. (2011). Loneliness Is Associated with Sleep Fragmentation in a Communal Society. Sleep.

[53] Riegel B., Weaver T.E. (2009). Poor sleep and impaired self-care: Towards a comprehensive model linking sleep, cognition, and heart failure outcomes. Eur. J. Cardiovasc. Nurs..

[54] Nebes R.D., Buysse D.J., Halligan E.M., Houck P.R., Monk T.H. (2009). Self-Reported Sleep Quality Predicts Poor Cognitive Performance in Healthy Older Adults. J. Gerontol. Ser. B Psychol. Sci. Soc. Sci..

[55] McHugh J.E., Casey A.M., Lawlor B.A. (2011). Psychosocial correlates of aspects of sleep quality in community-dwelling Irish older adults. Aging Ment. Health.

[56] Griffin S.C., Williams A.B., Ravyts S.G., Mladen S.N., Rybarczyk B.D. (2020). Loneliness and sleep: A systematic review and meta-analysis. Health Psychol. Open.

[57] Cacioppo J.T., Ernst J.M., Burleson M.H., McClintock M.K., Malarkey W.B., Hawkley L.C., Kowalewski R.B., Paulsen A., Hobson J., Hugdahl K. (2000). Lonely traits and concomitant physiological processes: The MacArthur social neuroscience studies. Int. J. Psychophysiol..

[58] Hawkley L.C., Thisted R.A., Cacioppo J.T. (2009). Loneliness predicts reduced physical activity: Cross-sectional & longitudinal analyses. Health Psychol..

[59] Donovan N.J., Wu Q., Rentz D.M., Sperling R.A., Marshall G.A., Glymour M.M. (2017). Loneliness, depression and cognitive function in older U.S. adults. Int. J. Geriatr. Psychiatry.

[60] Ertel K.A., Glymour M.M., Berkman L.F. (2008). Effects of Social Integration on Preserving Memory Function in a Nationally Representative US Elderly Population. Am. J. Public Health.

[61] Wallace D.L., Han M.-H., Graham D.L., Green T.A., Vialou V., Iñiguez S., Cao J.-L., Kirk A., Chakravarty S., Kumar A. (2009). CREB regulation of nucleus accumbens excitability mediates social isolation–induced behavioral deficits. Nat. Neurosci..

[62] Luchetti M., Terracciano A., Aschwanden D., Lee J.H., Stephan Y., Sutin A.R. (2020). Loneliness is associated with risk of cognitive impairment in the Survey of Health, Ageing and Retirement in Europe. Int. J. Geriatr. Psychiatry.

[63] Wilson R.S., Krueger K.R., Arnold S.E., Schneider J.A., Kelly J.F., Barnes L.L., Tang Y., Bennett D.A. (2007). Loneliness and Risk of Alzheimer Disease. Arch. Gen. Psychiatry.

[64] Yu B., Steptoe A., Chen Y., Jia X. (2021). Social isolation, rather than loneliness, is associated with cognitive decline in older adults: The China Health and Retirement Longitudinal Study. Psychol. Med..

[65] Stewart C.C., Yu L., Glover C.M., Mottola G., Bennett D.A., Wilson R.S., Boyle P.A. (2020). Loneliness Interacts With Cognition in Relation to Healthcare and Financial Decision Making Among Community-Dwelling Older Adults. Gerontologist.

[66] Tilvis R.S., Kähönen-Väre M.H., Jolkkonen J., Valvanne J., Pitkala K.H., Strandberg T.E. (2004). Predictors of Cognitive Decline and Mortality of Aged People Over a 10-Year Period. J. Gerontol. Ser. A Biol. Sci. Med. Sci..

[67] Akhter-Khan S.C., Tao Q., Ang T.F.A., Itchapurapu I.S., Alosco M.L., Mez J., Piers R.J., Steffens D.C., Au R., Qiu W.Q. (2021). Associations of loneliness with risk of Alzheimer’s disease dementia in the Framingham Heart Study. Alzheimer Dement..

[68] Sundström A., Adolfsson A.N., Nordin M., Adolfsson R. (2020). Loneliness Increases the Risk of All-Cause Dementia and Alzheimer’s Disease. J. Gerontol. Ser. B.

[69] Sutin A.R., Stephan Y., Luchetti M., Terracciano A. (2020). Loneliness and Risk of Dementia. J. Gerontol. Ser. B.

[70] Sun W., Matsuoka T., Oba H., Narumoto J. (2021). Importance of loneliness in behavioral and psychological symptoms of dementia. Int. J. Geriatr. Psychiatry.

[71] Krendl A.C., Perry B.L. (2021). The Impact of Sheltering in Place During the COVID-19 Pandemic on Older Adults’ Social and Mental Well-Being. J. Gerontol. Ser. B.

[72] Lee S.L., Pearce E., Ajnakina O., Johnson S., Lewis G., Mann F., Pitman A., Solmi F., Sommerlad A., Steptoe A. (2021). The association between loneliness and depressive symptoms among adults aged 50 years and older: A 12-year population-based cohort study. Lancet Psychiatry.

[73] McQuaid R.J., Cox S.M., Ogunlana A., Jaworska N. (2021). The burden of loneliness: Implications of the social determinants of health during COVID-19. Psychiatry Res..

[74] Rivera D.S., Lindsay C.B., Oliva C.A., Codocedo J.F., Bozinovic F., Inestrosa N.C. (2020). Effects of long-lasting social isolation and re-socialization on cognitive performance and brain activity: A longitudinal study in Octodon degus. Sci. Rep..

[75] van Roekel E., Ha T., Verhagen M., Kuntsche E., Scholte R.H.J., Engels R.C.M.E. (2015). Social stress in early adolescents’ daily lives: Associations with affect and loneliness. J. Adolesc..

[76] Campagne D.M. (2019). Stress and perceived social isolation (loneliness). Arch. Gerontol. Geriatr..

[77] Doane L.D., Adam E.K. (2010). Loneliness and cortisol: Momentary, day-to-day, and trait associations. Psychoneuroendocrinology.

[78] Steptoe A., Owen N., Kunz-Ebrecht S.R., Brydon L. (2004). Loneliness and neuroendocrine, cardiovascular, and inflammatory stress responses in middle-aged men and women. Psychoneuroendocrinology.

[79] Hawkley L.C., Cole S.W., Capitanio J.P., Norman G.J., Cacioppo J.T. (2012). Effects of social isolation on glucocorticoid regulation in social mammals. Horm. Behav..

[80] Aguilera G. (2011). HPA axis responsiveness to stress: Implications for healthy aging. Exp. Gerontol..

[81] Varghese F.P., Brown E.S. (2001). The Hypothalamic-Pituitary-Adrenal Axis in Major Depressive Disorder. Prim. Care Companion J. Clin. Psychiatry.

[82] Dunlavey C.J. (2018). Introduction to the Hypothalamic-Pituitary-Adrenal Axis: Healthy and Dysregulated Stress Responses, Developmental Stress and Neurodegeneration. J. Undergrad. Neurosci. Educ..

[83] Miller G.E., Chen E., Zhou E.S. (2007). If it goes up, must it come down? Chronic stress and the hypothalamic-pituitary-adrenocortical axis in humans. Psychol. Bull..

[84] Loades M.E., Chatburn E., Higson-Sweeney N., Reynolds S., Shafran R., Brigden A., Linney C., McManus M.N., Borwick C., Crawley E. (2020). Rapid Systematic Review: The Impact of Social Isolation and Loneliness on the Mental Health of Children and Adolescents in the Context of COVID-19. J. Am. Acad. Child Adolesc. Psychiatry.

[85] Lara E., Moreno-Agostino D., Martín-María N., Miret M., Rico-Uribe L.A., Olaya B., Cabello M., Haro J.M., Ayuso-Mateos J.L. (2020). Exploring the effect of loneliness on all-cause mortality: Are there differences between older adults and younger and middle-aged adults?. Soc. Sci. Med..

[86] Giné-Garriga M., Jerez-Roig J., Coll-Planas L., Skelton D.A., Inzitari M., Booth J., Souza D.L. (2021). Is loneliness a predictor of the modern geriatric giants? Analysis from the survey of health, ageing, and retirement in Europe. Maturitas.

[87] Beutel M.E., Klein E.M., Brähler E., Reiner I., Jünger C., Michal M., Wiltink J., Wild P.S., Münzel T., Lackner K.J. (2017). Loneliness in the general population: Prevalence, determinants and relations to mental health. BMC Psychiatry.

[88] Noback M., Zhang G., White N., Barrow J.C., Carr G.V. (2021). Post-weaning social isolation increases ΔFosB/FosB protein expression in sex-specific patterns in the prelimbic/infralimbic cortex and hippocampus in mice. Neurosci. Lett..

[89] Donovan M., Mackey C.S., Platt G.N., Rounds J., Brown A.N., Trickey D.J., Liu Y., Jones K.M., Wang Z. (2020). Social isolation alters behavior, the gut-immune-brain axis, and neurochemical circuits in male and female prairie voles. Neurobiol. Stress.

[90] Sol K., Sharifian N., Manly J.J., Brickman A.M., Zahodne L.B. (2021). Associations Between Loneliness, Reading Ability and Episodic Memory in Non-Hispanic Black and White Older Adults. Arch. Clin. Neuropsychol..

[91] Mann F., Bone J., Lloyd-Evans B., Frerichs J., Pinfold V., Ma R., Wang J., Johnson S. (2017). A life less lonely: The state of the art in interventions to reduce loneliness in people with mental health problems. Soc. Psychiatry Psychiatr. Epidemiol..

[92] Masi C.M., Chen H.-Y., Hawkley L.C., Cacioppo J.T. (2011). A Meta-Analysis of Interventions to Reduce Loneliness. Personal. Soc. Psychol. Rev..

[93] Shvedko A.V., Thompson J., Greig C.A., Whittaker A.C. (2018). Physical Activity Intervention for Loneliness (PAIL) in community-dwelling older adults: Protocol for a feasibility study. Pilot Feasibility Stud..

[94] Cohen G.D., Perlstein S., Chapline J., Kelly J., Firth K.M., Simmens S. (2006). The Impact of Professionally Conducted Cultural Programs on the Physical Health, Mental Health, and Social Functioning of Older Adults. Gerontologist.

[95] Fakoya O.A., McCorry N.K., Donnelly M. (2020). Loneliness and social isolation interventions for older adults: A scoping review of reviews. BMC Public Health.

